# Impact of Extrusion on Biofunctional, Rheological, Thermal, and Structural Properties of Corn Starch/Whey Protein Isolate Blends During In Vitro Gastrointestinal Digestion

**DOI:** 10.3390/polym17233211

**Published:** 2025-12-02

**Authors:** José A. Téllez-Morales, Jesús Rodríguez-Miranda, Fátima S. Serrano-Villa, Gustavo F. Gutiérrez-López, Reynold R. Farrera-Rebollo, Georgina Calderón-Domínguez

**Affiliations:** 1 Departamento de Ingeniería Bioquímica, Escuela Nacional de Ciencias Biológicas, Instituto Politécnico Nacional, Gustavo A. Madero, Mexico City 07738, Mexico; 2 Departamento de Ingeniería Química y Bioquímica, Instituto Tecnológico de Tuxtepec, Tecnológico Nacional de México, Tuxtepec 68350, Oaxaca, Mexico

**Keywords:** extrusion, extruded, biofunctionality, DSC, rheology, digestion, improving health properties of food by sharing our knowledge on the digestive process (INFOGEST)

## Abstract

This study examines the effects of extrusion cooking on the biofunctional, rheological, thermal, and structural properties of corn starch (CS)/whey protein isolate (WPI) blends (100/0, 50/50, 0/100 *w*/*w*, both raw and extruded) during in vitro gastrointestinal digestion. Extrusion and in vitro digestion increased antioxidant activity (2,2′-Azino-Bis (3-Ethylbenzothiazoline-6-Sulfonic Acid) Diammonium Salt and 2,2-Diphenyl-1-Picrylhydrazyl). Extrusion improved the bioaccessibility of angiotensin-converting enzyme (ACE-1) inhibitory peptides, leading to high inhibition (>90%) in the intestinal phase across all samples, with this effect consistent between raw and extruded samples during digestion. The in vitro digestion process changes the rheological behavior of the samples, from a non-Newtonian fluid (dilatant) to a Newtonian fluid. Notably, extruded CS maintained pseudoplastic behavior across all phases. Thermally, extrusion resulted in complete gelatinization of CS and denaturation of WPI, as evidenced by the absence of endotherms. Structurally, extrusion induced unfolding of WPI α-helix and β-sheet regions, leading to the formation of β-turns and random coils, which could enhance enzyme accessibility. For CS, a decrease in the degree of double helix and order was observed, indicating an alteration of its ordered molecular structure. Additionally, the extrusion process slightly increased the amount of resistant starch. This work shows that extrusion generates antioxidant compounds by bioactive peptide release.

## 1. Introduction

Understanding the impact of food structure and composition on health increasingly involves simulating digestion in the gastrointestinal tract. The applied protocol is based on an international consensus developed by the COST INFOGEST network (Improving health properties of food by sharing our knowledge on the digestive process). Using this method, food samples undergo sequential oral, gastric, and intestinal digestion. Parameters such as enzymes, electrolytes, dilution, bile, pH, and digestion time are based on available physiological data. Therefore, the method enables the evaluation of digestion results by analyzing digestion products, such as simple sugars, peptides/amino acids, and fatty acids [1,2]. Different enzymatic digestion processes produce different amounts and types of peptides in the same food matrix. The selection of an enzyme to catalyze protein hydrolysis depends on evaluating its selectivity and the literature indicating its applicability. The enzymatic hydrolysis of whey protein isolate (WPI) employs various proteases, each of which influences the composition and bioactivity of the resulting peptides [3,4]. Bioactive peptides are protein fragments that positively affect body functions and health. These peptides are inactive within proteins but can be released during enzymatic hydrolysis under specific gastrointestinal tract conditions. Bioactive peptides have antimicrobial, antithrombotic, antihypertensive, opioid, immunomodulatory, and antioxidant activities that depend on their amino acid sequence and composition [5]. The growing interest in healthy foods has created a market for snacks that satisfy basic dietary needs and position themselves as carriers of biologically active compounds. This has led to the development of functional foods adapted to current needs [6]. The most common process for producing these ready-to-eat foods is extrusion cooking, which is used for a wide range of products, including snacks. This thermomechanical process can modify the structure of starch, which is the main component responsible for product expansion. This modification occurs through stages of gelatinization, melting, and degradation [7,8,9,10]. Interactions between starch and protein contribute to lower starch digestibility. Currently, most research has focused on the impact of protein on starch structure and digestibility by conventional hydrothermal treatments, such as heat–moisture treatment, cooking, and annealing. However, how protein affects starch characteristics in current processing technologies, such as ultra-high pressure, microwaves, and extrusion, has not been adequately investigated [11]. De Boer et al. [12] demonstrated the potential of high-moisture extrusion to produce high-quality soybean extrudates by showing that amino acid values and in vitro protein digestibility can be maintained and improved under varying processing conditions; consequently, the interaction between corn starch and whey proteins during extrusion results in structural changes that improve the biofunctionality of extruded products. The primary objective of this research study was to conduct a comprehensive evaluation of the impact of extrusion cooking on the biofunctional, rheological, thermal, and structural properties of corn starch (CS) and whey protein isolate (WPI) blends, specifically during in vitro gastrointestinal digestion simulated using the standardized INFOGEST protocol.

## 2. Materials and Methods

### 2.1. Materials

Whey protein isolate (WPI) (86.2% protein, 1.07% ash, and 0.034% lipid) from Nature’s Best brand (Nature’s Best, Inc., Missoula, MT, USA) and corn starch (CS) (98% starch, 0.62% lipid and 0.49% ash) from IMSA brand (Industrializadora de Maíz. S. A de C.V., Guadalajara, Mexico) were used.

### 2.2. Extrusion Process

Raw corn starch/whey protein isolate mixtures were prepared in duplicate at a ratio of 100/0, 50/50, and 0/100 (*w*/*w*), according to Téllez-Morales et al. [13]. Extrusion cooking was carried out in a single-screw laboratory extruder (Brabender, Model AEV 330, Duisburg, Germany). Moisture content of the samples was adjusted to 20%, letting them rest in a plastic sealed bag for 14 h before being processed. Extrusion was carried out at a screw speed of 200 rpm, with a screw 1:1 compression ratio and a 6 mm outlet die. Extrusion temperatures were as follows: zone 1 = 100 °C, zone 2 = 120 °C, and zone 3 = 140 °C. Samples were dried at 60 °C for 2 h (Binder drying oven, Model ED 115, Duisburg, Germany) after leaving the extruder. Afterwards the dried samples were ground (Hamilton Beach, Model 80350R, Guangdong, China) to a particle size of 590 µm (No. 35 mesh sieve) and stored at room temperature (25 °C) in sealed bags for analyses, which were performed during the same week of sample production. This sample obtention procedure was carefully followed to maintain process conditions. Independent duplicate samples (500 g) of each extruded formulation were obtained. All process conditions were based on those reported by Téllez-Morales et al. [13] for the same materials.

### 2.3. In Vitro Gastrointestinal Digestion by INFOGEST

**Figure 1:**
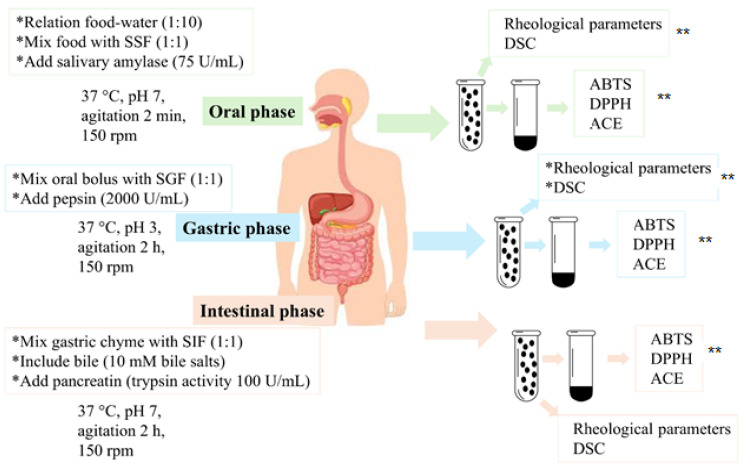
Simulated in vitro gastrointestinal procedure for raw and extruded samples using INFOGEST. SSF: simulated oral salt fluid solution; SGF: simulated gastric saline fluid solution; SIF: simulated intestinal fluid solution; * Chemical solutions added, enzymes used and experimental conditions employed during the three digestion phases (oral, gastric, intestinal); ** Analyses carried out.

The in vitro digestion procedure was performed according to the standardized protocol developed by the INFOGEST network [1], which consists of simulating the digestion process by adding salts and enzymes characteristic of the oral, gastric, and duodenal cavities. Briefly (Figure 1), to simulate the oral phase, 0.30 g of sample was mixed in 3 mL of water, and 2.4 mL of simulated oral fluid salt solution (SSF) was added. An amount of 15 µL of CaCl_2_ (H_2_O)_2_ 0.3 M, 285 µL of water, and 300 µL of porcine α-amylase (16 u/mg; A3176-500KU, Sigma-Aldrich, St. Louis, MO, USA) were added to each sample, and the pH 7 of the sample was checked. Samples were placed in an incubator (Barnstead International, model SKA4000-7, Dubuque, IA, USA) and shaken at 150 rpm for 2 min and 37 °C. To simulate the gastric phase, 4.8 mL of simulated gastric saline (SGF) was added to the oral phase bolus. For each sample, 3 µL of 0.3 M CaCl_2_ (H_2_O)_2_, 897 µL of water, 300 µL of porcine pepsin (3640 u/mg; P7000-25G, Sigma-Aldrich, St. Louis, MO, USA), and 6 M HCl were added to reduce the pH of the simulated gastric fluid to 3 with a digestion time of 2 h (37 °C −150 rpm). To simulate the intestinal phase of digestion, the gastric chyme obtained from the gastric simulation was mixed with 5.1 mL of simulated intestinal fluid solution (SIF). An amount of 24 µL CaCl_2_ (H_2_O)_2_ 0.3 M, 2.38 mL water, 1.5 mL bile salts (approximately 50% sodium cholate and 50% sodium deoxycholate; B8756-50G, Sigma-Aldrich), and 3 mL porcine pancreatin (100 u/mg; P7545-25G, Sigma-Aldrich, St. Louis, MO, USA) were added. The pH of the solution was adjusted with 1 M NaOH to neutralize the mixture to pH 7, and it was kept in incubation for 2 h (37 °C −150 rpm), the samples were kept frozen until further analyses.

### 2.4. Rheological Parameters

To measure the viscosity behavior at different shear rates, 0.30 g of samples was dissolved in 3 mL of distilled water, and 1500 µL of the prepared samples was placed in the sample holder of the rheometer (TA Instruments, Discovery HR-3, New Castle, DE, USA). A 40 mm Peltier parallel steel plate (998807) was used with a gap of 1000 µm, constant 37 °C temperature, and a shear rate that was increased from 2.5 to 63 s^−1^. The same method was used for the samples of the different phases of digestion, with 1500 µL used directly. The rheometer software (TA Instruments TRIOS, version 4.0.2.30774, New Castle, DE, USA) was used to adjust a mathematical model to the data, with the best fit in all cases being the Herschel–Bulkley model, as shown in Equation (1), because the R^2^ value of all the other models tested by the software were even lower. Furthermore, this yield stress model incorporates the elements of Newtonian, Power law, and Bingham fluid models.

σ = σ_y_ + k γ^n^ (1)

with σ_y_ the yield stress, k the consistency and n the power law index.

For σ_y_ = 0 and n = 1 => Newtonian fluid.

For σ_y_ = 0 and n ≠ 1 => Power Law fluid.

For σ_y_ ≠ 0 and n = 1 => Bingham fluid.

The value of k is considered a measurement of the fluid’s consistency; the larger the value of k, the more viscous the fluid will be. The value of n is a measurement of the degree of non-Newtonian behavior. The farther “n” deviates from unity, the more pronounced the fluid’s non-Newtonian properties are [14].

### 2.5. Antioxidant Activity by ABTS (2,2′-Azino-Bis (3-Ethylbenzothiazoline-6-Sulfonic Acid) Diammonium Salt)

The methodology of Leite et al. [15] was followed with modifications; ABTS radicals were obtained by reacting 7 mM (19.20 mg/5 mL water) ABTS stock solution (Sigma-Aldrich, 10102946001) with 2.45 mM (3.31 mg/5 mL water) potassium persulfate (Sigma-Aldrich, 216224) in the dark at room temperature (25 °C) for 12–16 h prior to use. The ABTS solution was diluted with distilled water to an absorbance of 0.700 at 754 nm. An amount of 50 µL of each sample was added to 250 µL of diluted ABTS solution. For the extract, 1 g of sample was weighed in 10 mL of water and sonicated for 30 min; in the case of the digestion phase samples, they were directly centrifuged (3060× *g* for 10 min) for analysis. The absorbances were measured after 6 min at 754 nm in a microplate reader (Thermo Fisher Scientific Multiskan Go, Waltham, MA, USA). Results were expressed in μM Trolox equivalents using a Trolox calibration curve at 1000 µM.

### 2.6. Antioxidant Activity by DPPH (2,2-Diphenyl-1-Picrylhydrazyl)

The inhibition potential of the 2,2-diphenyl-1-picrylhydrazyl radical (DPPH) was determined according to the methodology of Bobo-García et al. [16]. The preparation of the extract is as follows: 1 g of sample was weighed in 10 mL of water and sonicated for 30 min. In the case of the digestion phase samples, they were directly centrifuged (3060× *g* for 10 min) for analysis. An amount of 180 µL of DPPH methanolic solution (150 µM) was added to 20 µL of each sample in a microplate, then was mixed and allowed to stand in the dark for 40 min. The absorbance of the reactions was read at 515 nm on a microplate reader (Thermo Fisher Scientific Multiskan Go, Waltham, MA, USA). Results were expressed in μM Trolox equivalents using a Trolox calibration curve at 1000 µM.

### 2.7. ACE-I Activity Inhibition

The ACE inhibitory activity was determined using the ACE Kit- (DOJINDO ACE Kit-WST: A502 assay, Rockville, MD, USA). This assay is based on the mechanism of action of angiotensin-converting enzyme (ACE), which converts angiotensin I to angiotensin II. The assay determines the amount of 3-hydroxybutyric acid produced. In this case, the raw and extruded samples were dissolved in HPLC grade water at a concentration of 1% *w*/*v* and centrifuged at 3060× *g* for 10 min, and the supernatants were used for analyses, while samples from the different digestion steps were directly centrifuged using the supernatant as well.

### 2.8. Thermal Properties by DSC

The thermal characteristics of the samples were studied using a differential scanning calorimeter (DSC Q2000, TA Instruments Waters, Newcastle, DE, USA) following the manufacturer’s methodology for calibration. In the undigested raw samples, approximately 5 mg was weighed with the moisture adjusted to 20%. In order to simulate the temperature and pressure extrusion conditions, in a 40 μL aluminum hermetic pan, a heating sweep of 10 °C/min up to 145 °C was used. On the other hand, in digestion, the samples (13 µL) were loaded into aluminum hermetic pans of 40 μL capacity, the samples were hermetically sealed, and an empty pan was used as reference. The sample pans were heated at a rate of 5 °C/min from 25 to 110 °C. The thermal transition of the samples was defined as To (onset temperature), Tp (peak gelatinization temperature), Tc (conclusion temperature), and ∆H, referred to as the gelatinization enthalpy. The percentage of gelatinization or degree of gelatinization (DG) was calculated in relation to the extruded sample using Equation (2) [17].

DG = (1 − (∆H extruded sample)/(∆H raw sample)) × 100(2)

### 2.9. Concentration of Secondary Proteins in WPI and the Degree of Order and Double Helix in the Fingerprint of Corn Starch by FTIR

The procedure for obtaining the FTIR spectra is detailed in a previous publication, as is the wavelength profiles of the respective raw and extruded samples [8]. In this case, the amide I band of WPI in the obtained spectra, which ranges from 1600 to 1700 cm^−1^, was analyzed for fractions of secondary structures. These fractions were calculated using a Gaussian function with OriginPro software, version 10.1.0.178 (OriginLab Corporation, Northampton, MA, USA). Previously, baseline fitting and subsequent measurement were performed at respective wavelengths for the β-sheet (1610–1640 cm^−1^), random coil (1640–1650 cm^−1^), α-helix (1650–1664 cm^−1^), and β-turn (1664–1695 cm^−1^) structures [18]. For corn starch, the degree of the double helix was measured by dividing the intensity ratio at 995/1022 cm^−1^ and the degree of order by dividing the intensity ratio at 1047/1022 cm^−1^ of the raw and extruded samples after adjusting the baseline [19].

### 2.10. Determination of Resistant, Digestible, and Total Starch

The analysis was performed using the resistant starch kit (Megazyme K-RSTAR 05/19, Wicklow, Ireland), which involves incubating the samples with pancreatic α-amylase and amyloglucosidase (AMG) in agitation for 16 h at 37 °C. During this time, the non-resistant or digestible starch is solubilized and hydrolyzed into D-glucose by the combined action of the two enzymes.

### 2.11. Statistical Analysis

The results were examined statistically (mean ± standard deviation), and the significant difference was studied by one-way analysis of variance at 95% confidence level using Fisher’s Least Difference Test using OriginPro software, version 10.1.0.178, (OriginLab Corporation, Northampton, MA, USA).

## 3. Results and Discussion

### 3.1. Rheological Parameters

Table 1 shows the Herschel–Bulkley model parameters for the raw, extruded, and gastrointestinal digestion samples. This model was the best fit for the experimental data due to its R^2^ value, which was greater than 0.95 in most cases, except for extrudates without digestion (WPI 0%, 50%, 100%), and the WPI 0% extrudate in the gastric phase. From the data, it can be observed that none of the samples require yield stress (σ_y_) to begin flowing; therefore, they are not Bingham plastic fluids. Additionally, it can be seen that prior to digestion, most of the samples exhibit a flow index (n) that is slightly dilatant (1.11 to 1.21). This means their viscosity increases as shear stress increases. This behavior is observed in materials such as starch suspensions in water and solid dispersions in liquids [20]. Given that n is slightly above 1, the dilatant effect is minimal. Kumar et al. [21] reported that replacing oat starch paste with milk proteins (whey protein concentrate and whey lactalbumin) and skim milk powder decreased the system’s viscosity compared to oat starch alone. Despite this reduction, the blends maintained pseudoplastic behavior, in which the apparent viscosity decreases with shear rate. Extruded corn starch was the only sample that maintained pseudoplastic behavior in all phases. This property is important in many food products because pseudoplastic materials have suspension properties at low shear rates and low viscosities at high shear rates [22]. However, extrusion has been reported to destabilize the starch structure, causing its gelatinization and degradation and making it more susceptible to enzymatic digestion [11]. During the oral phase, α-amylase partially hydrolyzes starch, inhibiting the formation of semi-rigid structures in the fluid matrix and promoting a shift from slightly dilatant to Newtonian behavior. In the gastric phase, acidification causes proteins to denature and aggregate, altering solution interactions and producing a more structured system resistant to flow. While CS is less directly impacted by acidic pH, it may still experience changes in solubility and its capacity to interact with WPI. Pepsin further acts on WPI, generating fragments that have altered solubility and interaction properties [4]. The presence of high concentrations of suspended solids can cause a decrease in viscosity at higher shear stresses, which is characteristic of pseudoplasticity [20], observed during the gastric phase. During the intestinal phase, degradation creates smaller, less cohesive structures that reduce the tendency toward pseudoplastic behavior [4]. This is due to the action of pancreatic amylase, protease (trypsin and chymotrypsin), ribonuclease, and lipase enzymes, which further hydrolyze corn starch and whey protein isolate. The reduction in molecule size decreases resistance to deformation, which can lead to Newtonian fluid behavior, as observed.

### 3.2. Antioxidant Activity by ABTS and DPPH

**Figure 2:**
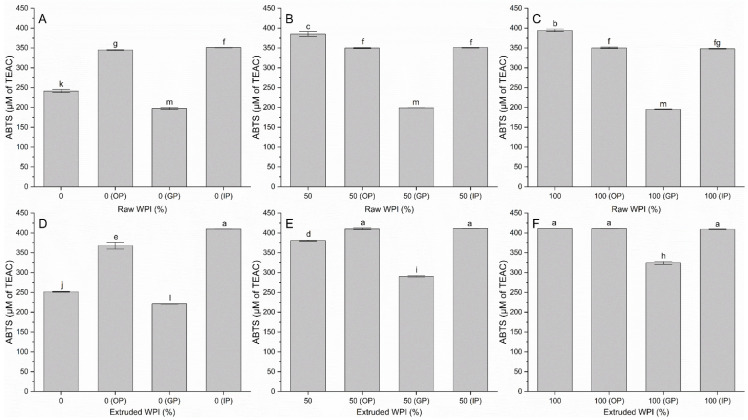
Antioxidant activity by ABTS in raw, extruded, and in vitro gastrointestinal digestion samples. WPI: whey protein isolate, the differences in percentage in the samples correspond to corn starch. OP: oral phase, GP: gastric phase, IP: intestinal phase. (**A**) Raw WPI (0%), (**B**) Raw WPI (50%), (**C**) Raw WPI (100%), (**D**) Extruded WPI (0%), (**E**) Extruded WPI (50%), (**F**) Extruded WPI (100%). Columns with different lowercase letters are significantly different (*p* < 0.05).

**Figure 3:**
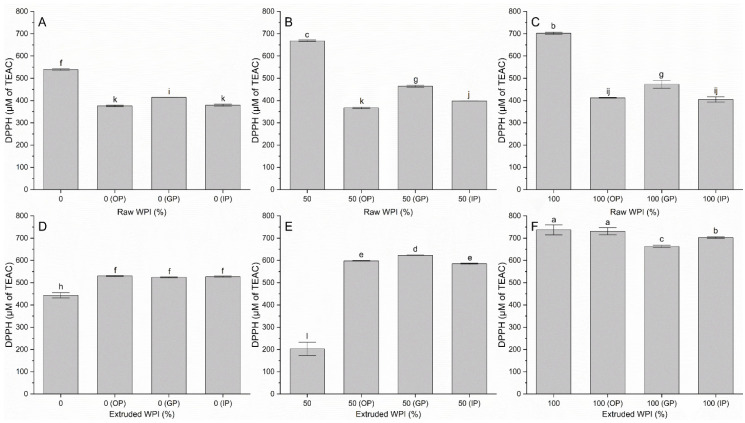
Antioxidant activity by DPPH in raw, extruded, and in vitro gastrointestinal digestion samples. WPI: whey protein isolate, the differences in percentage in the samples correspond to corn starch. OP: oral phase, GP: gastric phase, IP: intestinal phase. (**A**) Raw WPI (0%), (**B**) Raw WPI (50%), (**C**) Raw WPI (100%), (**D**) Extruded WPI (0%), (**E**) Extruded WPI (50%), (**F**) Extruded WPI (100%). Columns with different lowercase letters are significantly different (*p* < 0.05).

ABTS and DPPH assays were used to measure the antioxidant capacity of raw and extruded WPI as well as its capacity after digestion. Figure 2 shows the data of the antioxidant activity by ABTS of the raw and extruded samples, and Figure 3 shows the antioxidant activity by DPPH. It can be observed that the extrusion showed varied results. It is worth to highlight that the extruded WPI presented the highest statistically different antioxidant capacity. This is due to the generation of bioactive peptides during the extrusion process, caused by the combination of high temperature, shear, and pressure. These three parameters cause the denaturation and fragmentation of proteins, which facilitates the release of antioxidant peptides that can contribute to the neutralization of free radicals [3,23]. CS itself is not a primary antioxidant; however, some studies suggest that polysaccharides may exhibit antioxidant activity through mechanisms such as hydrogen donation or metal chelation. Nonetheless, this contribution is usually less than that of phenolic compounds and carotenoids [24,25]. High extrusion temperatures, shearing, and pressure may promote Maillard reactions (between amino groups of WPI and reducing sugars of CS) and caramelization of sugars. The products of these reactions are known to possess reducing properties and, therefore, could contribute to the higher antioxidant activity observed in the samples [26,27]. The presence of carbohydrates or phenolic compounds in the extruded matrix may affect antioxidant activity through synergism or free radical stabilization [27]. Xu et al. [28] reported that despite the overall decrease caused by extrusion, the addition of an enzyme in enzymatic extrusion improved antioxidant capacity. Furthermore, both DPPH and ABTS values increased significantly with increasing soybean content in the blends between 15 and 75%. In the simulation of the digestion, a significant decrease in the gastric phase by ABTS is observed in all samples, possibly due to the action of pepsin and the acidic environment that can break peptide bonds, reducing the concentration of peptides with antioxidant activity; in addition, the ABTS method is more sensitive to water-soluble antioxidants, and some peptides generated in the gastric digestion could become less soluble, affecting the measurement [3]. In contrast, DPPH digestion reveals no uniform behavior. However, there is more antioxidant activity with a statistical difference in the extruded samples during the three phases of digestion. This is because extrusion can modify the structure of components, facilitating the release of antioxidant compounds during digestion. Additionally, products generated during extrusion can react further in the acidic gastric environment, forming compounds with higher antioxidant capacity. During digestion, enzymes fragment proteins and carbohydrates, generating peptides and other compounds with antioxidant activity. pH variation and the presence of bile salts can also modify the solubility and reactivity of the antioxidants in extrudates. Sarmiento-Torres et al. [6] found that extrusion alone did not significantly affect ABTS and DPPH activity prior to digestion. However, the in vitro digestion process reduced ABTS and DPPH values in both raw and extruded samples. When considering the combined effect of extrusion followed by digestion, the digested extrudates exhibited significantly lower antioxidant activity and bioaccessibility compared to the digested raw samples. This post-digestion reduction was likely due to extrusion-induced changes in phenolic compounds. The observed differences in antioxidant activity can be attributed to the specificity of each enzyme to a particular site in the peptide chain during digestion. Furthermore, this phenomenon is associated with the degree of hydrolysis, which ultimately results in hydrolysates with different amino acid compositions. Antioxidant activity is also attributed to easily oxidizable amino acid groups, such as those found in tryptophan, histidine, cysteine, tyrosine, and methionine [3]. Some amino acid residues, such as aromatic and sulfur residues, have been reported to contribute to the antioxidant activity of proteins due to their ability to donate protons to reactive free radicals [18]. In summary, extrusion had a significant positive effect on antioxidant activity in most samples; however, simulated digestion did as well.

### 3.3. ACE-1 Activity Inhibition

**Figure 4:**
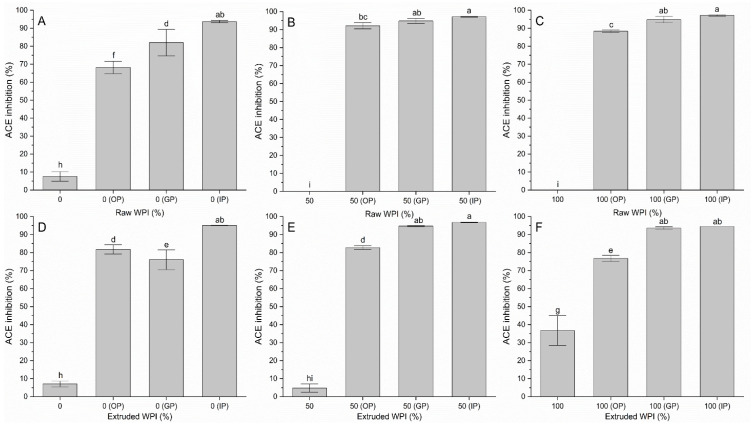
ACE-1 activity inhibition in raw, extruded, and in vitro gastrointestinal digestion samples. WPI: whey protein isolate, the differences in percentage in the samples correspond to corn starch. OP: oral phase, GP: gastric phase, IP: intestinal phase. (**A**) Raw WPI (0%), (**B**) Raw WPI (50%), (**C**) Raw WPI (100%), (**D**) Extruded WPI (0%), (**E**) Extruded WPI (50%), (**F**) Extruded WPI (100%). Columns with different lowercase letters are significantly different (*p* < 0.05).

Figure 4 shows the inhibition of angiotensin-converting enzyme 1 (ACE-1). It is clear that extrusion positively affected inhibition, highlighting the effect of WPI enhanced by extrusion. This is due to the generation of bioactive peptides during the process. This thermomechanical treatment causes denaturation and fragmentation of proteins, releasing peptides with inhibitory activity on ACE-1 [29]. Some of these peptides from α-lactoalbumin and β-lactoglobulin, have been shown to effectively reduce blood pressure. They do so by blocking the action of ACE-1 and preventing the conversion of angiotensin I to angiotensin II, a potent vasoconstrictor [29,30]. A significant increase in ACE-1 inhibition was observed at different stages of digestion for all raw and extruded samples. In the intestinal phase, inhibition exceeded 90% without showing statistical differences. Although some proteins in corn are present in low amounts in purified starch, they can generate inhibitory peptides when digested. These peptides can be released or become more accessible after the action of digestive enzymes during simulation. Additionally, the products of corn starch digestion could directly affect ACE-1. Both Czelej et al. [3] and Rodriguez-Hernandez et al. [30] noted that the gastrointestinal enzymes responsible for producing biopeptides are pepsin, trypsin, chymotrypsin, and pancreatin. Therefore, it was anticipated that the in vitro simulation would result in the production of ACE-1 inhibitory peptides, as demonstrated by the findings. In summary, extrusion can improve the bioaccessibility of these peptides, facilitating their absorption in the digestive tract. However, this effect is the same for raw samples and digested samples, and extrusion does not impair their release.

### 3.4. Thermal Properties by DSC

**Figure 5:**
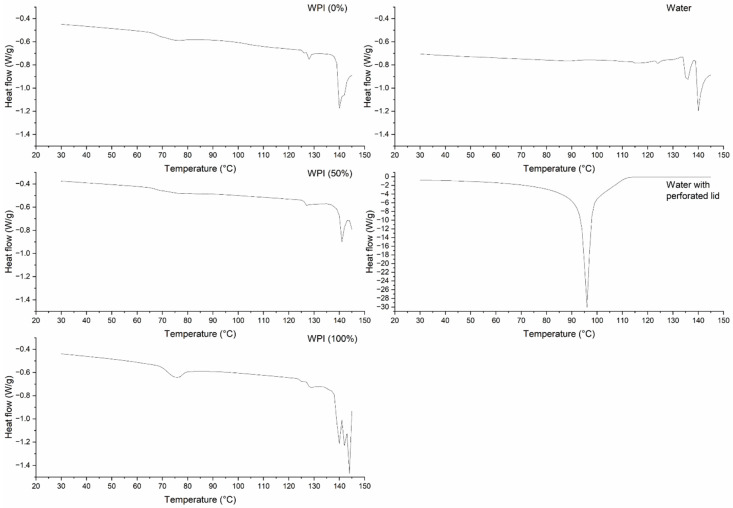
Simulation of temperature and pressure during extrusion of raw samples adjusted to 20% moisture content by DSC.

Figure 5 shows the endothermic behavior of the raw samples adjusted to a moisture content of 20%. With the extrusion simulation, changes in structure occur in the three samples between 70 and 80 °C. This is slightly higher than what was previously reported [8]; this increment in temperature for the endothermic transition could be attributed to the low initial moisture content due to the reduced availability of water for gelatinization and denaturation [31]. However, it is evident that temperature and pressure, independently of moisture content in extrusion, cause gelatinization of the CS and denaturation of the WPI. Also, the endothermic transitions reported above 120 °C are attributed to water. This increases the pressure and swells the pan lid, as was observed in the reference sample. This evidence shows that changes in starch and protein occur at temperatures below 90 °C.

Table 2 shows the thermal property results of gastrointestinal digestion. The data without digestion were previously reported and can be found in Téllez-Morales et al. [8]. The extruded samples follow the same trend. It can be concluded that the extruded samples did not undergo any structural changes during digestion because the CS granules were gelatinized and the WPI was denatured by extrusion cooking, not enzymatic action [8]. On the other hand, the effect of digestive enzymes is evident, starting with α-amylase, which hydrolyzes the CS in the oral phase in conjunction with salivary fluid. During the other two stages, pepsin and pancreatin, in conjunction with gastric and intestinal solutions, respectively, finish hydrolyzing the CS, especially the WPI [1]. This causes the loss of CS granules and the native structure of WPI in the raw samples, decreasing its endotherms and ∆H statistically until they disappear during digestive stages. Complete gelatinization and denaturation are observed.

### 3.5. FTIR Analysis

Our previous study [8] reported FTIR spectra; however, Table 3 shows the ratio of secondary structure content for the raw and extruded WPI of the amide I region. As previously reported [8], a significant decrease in the area under the curve was found compared to its extruded counterpart, which is indicative of a rearrangement in the secondary structure of the proteins. The changes generated by extrusion in the WPI cause unfolding in the α-helix and β-sheet regions, resulting in the formation of β-turns and random coil. These changes could be due to the disruption of hydrogen bonds that maintain the secondary structure of proteins [18,32,33]. The β-turns allow the protein to fold back on itself and stabilize it by connecting the ends via hydrogen bonds [33]. Similar to this study, Meng et al. [18] reported an α-helix concentration of 23.08% for raw WPI. Liang et al. [34] explained that an increase in α-helices and a decrease in β-sheets could indicate loosening of the protein molecule and exposure of more peptide bonds. This would make the molecule more accessible to digestive enzymes. Furthermore, these changes in secondary structure could be attributed to the disruption of interaction between molecules and the relative position of the induced local amino acid sequence. On the other hand, in the case of CS, a decrease in the double helix degree and degree of order is observed with extrusion in both starch and its mixture. This suggests an alteration of the ordered molecular structure on a short-range scale [19,31]. Marta et al. [35] proposed that the decrease in the degree of order is due to high temperatures and pressures causing significant structural damage—precisely what the thermomechanical extrusion process does. The stable and ordered structure of starch in extruded complexes can resist digestion by amylase to some extent [11]. These changes coincide with the decrease in crystallinity reported by XRD in our previous study on extrusion [8]. In summary, as reported [8], the FTIR analysis reveals that extrusion induces a physical destructuring mechanism rather than a covalent chemical modification. The decrease in intensity and characteristic broadening in the Amide I region evidences the mechanical unfolding of protein chains (loss of native structure). Simultaneously, the reduction in intensity in the 1047–1016 cm^−1^ region confirms the loss of crystallinity associated with the destruction of the starch grain. These structural changes imply a reorganization of the hydrogen bond network (3304 cm^−1^), consistent with the transition to a more amorphous and thermodynamically distinct phase.

### 3.6. Resistant, Digestible, and Total Starch

According to Table 4, the concentration of resistant starch in corn starch and its raw mixture is too low. This is mainly due to the simple molecular structure of starch and how it is processed or prepared, which makes it readily accessible to digestive enzymes in the human gastrointestinal tract [36]. Therefore, corn starch is easily digested and has a purity of ~98%, similar to the purity reported on the manufacturer’s label. On the other hand, the extrusion process slightly increased the amount of resistant starch by a statistically significant amount without affecting the mixture. Extrusion has been reported to increase the amount of resistant starch, specifically type 3, by converting native starch into a thermally more stable form. High shear combined with heat facilitates starch gelatinization, which is followed by retrogradation upon cooling. This process is important for the formation of resistant starch [37]. These statements are consistent with the significant decrease in the intensity of the peaks in the XRD diffractograms and the equally significant decrease in the viscosity profile by RVA previously reported in Téllez-Morales et al. [8]. In general, it has been reported that the interaction between amylose and amylopectin branches stabilizes the crystalline region after retrogradation. Competition with other components, such as proteins or lipids, could theoretically limit the availability of starch chains to form the crystalline double helices necessary for type 3 resistant starch [37].

## 4. Conclusions

The combination of factors in the rheological transition during gastrointestinal digestion is crucial because these transitions alter the fluid behavior of the oral bolus and gastric chyme. Thus, these transitions are important for optimizing nutrient bioavailability and absorption in the small intestine. Additionally, extrusion and simulated digestion both significantly increased antioxidant activity, as measured by ABTS and DPPH. Extrusion improves the bioaccessibility of ACE-1 inhibitory peptides, facilitating their absorption in the digestive tract. The raw samples undergoing digestion show a similar effect. In the DSC, the extruded samples follow the same trend: there are no endotherms in any of the samples at the different digestive stages. However, digestive enzymes cause the loss of CS granules and the native structure of WPI in the raw samples. This decreases their endotherms and ∆H until they disappear during digestion. This results in complete gelatinization and denaturation. Additionally, the endothermic transitions reported above 120 °C in the raw samples are attributed to water. In the FTIR results, extrusion in the WPI causes changes in secondary structure, while the CS shows decreased double helix and ordered structure. On the other hand, the extrusion process slightly increased the amount of resistant starch through gelatinization followed by retrogradation.

## Figures and Tables

**Table 1:** Herschel–Bulkley model parameters in raw and extruded samples and in their in vitro gastrointestinal digestion.

Parameters	Raw WPI 0%	Extruded WPI 0%	Raw WPI 50%	Extruded WPI 50%	Raw WPI 100%	Extruded WPI 100%
σ_y_ (Mpa)	1.32 × 10^−9^ ± 6.79 × 10^−10^	2.13 × 10^−4^ ± 3.88 × 10^−4^	1.03 × 10^−9^ ± 3.19 × 10^−9^	4.15 × 10^−6^ ± 2.36 × 10^−6^	4.09 × 10^−9^ ± 3.17 × 10^−9^	1.11 × 10^−5^ ± 1.64 × 10^−5^
k (Pa.s)	5.011 × 10^−4^ ± 2.31 × 10^−4^	2.48 × 10^2^ ± 3.61 × 10^2^	3.27 × 10^−3^ ± 4.50 × 10^−3^	1.04 × 10^−1^ ± 9.26 × 10^−2^	9.09 × 10^−4^ ± 3.89 × 10^−4^	1.69 × 10 ± 2.37 × 10
n	1.12 ± 0.14	0.59 ± 0.81	0.89 ± 0.35	1.21 ± 0.68	1.11 ± 0.10	1.12 ± 0.09
R^2^	0.999 ± 0.001	0.928 ± 0.042	0.989 ± 0.017	0.940 ± 0.090	0.998 ± 0.002	0.465 ± 0.512
Oral phase						
σ_y_ (Mpa)	2.89 × 10^−10^ ± 8.08 × 10^−10^	3.17 × 10^−7^ ± 5.77 × 10^−7^	1.56 × 10^−9^ ± 5.32 × 10^−10^	8.79 × 10^−10^ ± 3.24 × 10^−9^	2.21 × 10^−9^ ± 2.95 × 10^−9^	4.93 × 10^−9^ ± 1.09 × 10^−8^
k (Pa.s)	9.64 × 10^−4^ ± 5.95 × 10^−4^	3.00 × 10^−1^ ± 5.04 × 10^−1^	8.49 × 10^−4^ ± 4.86 × 10^−4^	5.83 × 10^−3^ ± 1.76 × 10^−3^	7.28 × 10^−4^ ± 3.01 × 10^−4^	3.52 × 10^−3^ ± 1.99 × 10^−3^
n	1.00 ± 0.14	0.57 ± 0.48	1.00 ± 0.11	0.72 ± 0.06	1.03 ± 0.11	0.76 ± 0.15
R^2^	0.998 ± 0.001	0.983 ± 0.016	0.998 ± 0.001	0.999 ± 0.001	0.997 ± 0.002	0.993 ± 0.009
Gastric phase						
σ_y_ (Mpa)	2.38 × 10^−9^ ± 2.27 × 10^−9^	1.57 × 10^−7^ ± 1.15 × 10^−6^	2.09 × 10^−9^ ± 1.31 × 10^−9^	2.81 × 10^−8^ ± 5.72 × 10^−8^	6.59 × 10^−10^ ± 3.47 × 10^−10^	3.81 × 10^−8^ ± 6.52 × 10^−8^
k (Pa.s)	2.27 × 10^−3^ ± 1.57 × 10^−3^	4.31 × 10^−1^ ± 6.05 × 10^−1^	7.63 × 10^−4^ ± 3.14 × 10^−4^	3.87 × 10^−3^ ± 5.33 × 10^−3^	1.10 × 10^−3^ ± 2.67 × 10^−4^	4.22 × 10^−2^ ± 5.44 × 10^−2^
n	0.80 ± 0.18	0.85 ± 0.89	0.97 ± 0.12	2.61 ± 3.26	0.89 ± 0.04	0.43 ± 0.38
R^2^	0.997 ± 0.001	0.837 ± 0.117	0.996 ± 0.005	0.961 ± 0.062	0.998 ± 0.003	0.959 ± 0.026
Intestinal phase						
σ_y_ (Mpa)	1.38 × 10^−9^ ± 1.09 × 10^−9^	6.51 × 10^−10^ ± 1.25 × 10^−9^	9.03 × 10^−10^ ± 7.38 × 10^−10^	1.22 × 10^−9^ ± 9.33 × 10^−10^	9.48 × 10^−5^ ± 1.64 × 10^−4^	1.45 × 10^−10^ ± 3.31 × 10^−10^
k (Pa.s)	6.80 × 10^−4^ ± 1.06 × 10^−4^	1.26 × 10^−3^ ± 3.68 × 10^−4^	5.27 × 10^−4^ ± 1.10 × 10^−4^	6.96 × 10^−4^ ± 2.10 × 10^−4^	9.48 × 10 ± 1.64 × 10^2^	7.40 × 10^−4^ ± 9.58 × 10^−5^
n	1.02 ± 0.04	0.88 ± 0.06	1.09 ± 0.05	1.04 ± 0.08	0.23 ± 0.20	0.99 ± 0.03
R^2^	0.996 ± 0.003	0.998 ± 0.002	0.999 ± 0.001	0.997 ± 0.001	0.998 ± 0.002	0.999 ± 0.002

σ_y_ (yield stress), k (consistency), n (power law index). WPI: whey protein isolate, the differences in percentage in the samples correspond to corn starch.

**Table 2:** Thermal properties of raw and extruded samples upon in vitro gastrointestinal digestion.

Parameters	Raw WPI 0%	Extruded WPI 0%	Raw WPI 50%	Extruded WPI 50%	Raw WPI 100%	Extruded WPI 100%
Oral phase						
T_o_ (°C)	65.55 ± 0.322 ^a^	--	66.87 ± 1.421 ^a^	--	--	--
T_p_ (°C)	72.03 ± 0.146 ^a^	--	72.99 ± 1.110 ^a^	--	--	--
T_c_ (°C)	88.29 ± 0.580 ^a^	--	84.49 ± 1.598 ^b^	--	--	--
∆H (J/g)	0.42 ± 0.094 ^a^	--	0.12 ± 0.039 ^b^	--	--	--
Gelatinization (%)	100 ± 0.000 ^a^	--	100 ± 0.000 ^a^	--	--	--
Gastric phase						
T_o_ (°C)	69.09 ± 0.159	--	--	--	--	--
T_p_ (°C)	75.17 ± 0.160	--	--	--	--	--
T_c_ (°C)	86.55 ± 0.634	--	--	--	--	--
∆H (J/g)	0.30 ± 0.045	--	--	--	--	--
Gelatinization (%)	100 ± 0.000	--	--	--	--	--

Onset temperature (T_o_), Peak temperature (T_p_), Conclusion temperature (T_c_), Enthalpy of gelatinization/denaturation (∆H). Values are mean ± standard deviation. Results with different superscripts in the same row mean that there is statistical difference (*p* < 0.05). WPI: whey protein isolate, the differences in percentage in the samples correspond to corn starch. Note: in the intestinal phase there is no more data to show.

**Table 3:** The concentration of secondary proteins in raw and extruded WPI in the Amide I region; in addition, the degree of order and double helix in the fingerprint of raw and extruded corn starch by FTIR.

Parameters (For WPI)	Raw WPI 50%	Extruded WPI 50%	Area Reduction (%)	Raw WPI 100%	Extruded WPI 100%	Area Reduction (%)
β-sheet (%) (1610–1640 cm^−1^)	38.72	38.33	19.14	31.91	35.48	44.37
Random coil (%) (1640–1650 cm^−1^)	11.52	10.37	26.50	8.10	13.09	19.15
α-helix (%) (1650–1664 cm^−1^)	23.57	23.57	18.35	22.67	21.74	52.02
β-turn (%) (1664–1695 cm^−1^)	26.19	27.73	13.52	37.32	29.70	60.18
**Parameters (For CS)**	**Raw WPI 0%**	**Extruded WPI 0%**	**Raw WPI 50%**	**Extruded WPI 50%**		
Double helix degree (995/1022 cm^−1^)	1.22	1.14	1.17	1.04		
Degree of order (1047/1022 cm^−1^)	0.65	0.62	0.70	0.61		

WPI: whey protein isolate, the differences in percentage in the samples correspond to corn starch.

**Table 4:** Concentration of resistant, digestible, and total starch in raw and extruded samples.

Parameters (g/100 g dwb)	Raw WPI 0%	Extruded WPI 0%	Raw WPI 50%	Extruded WPI 50%
Resistant starch	0.08 ± 0.018 ^c^	0.49 ± 0.098 ^a^	0.26 ± 0.073 ^b^	0.25 ± 0.076 ^b^
Digestible starch	98.67 ± 2.496 ^a^	92.12 ± 1.612 ^b^	42.51 ± 0.588 ^d^	45.95 ± 2.656 ^c^
Total starch	98.75 ± 2.504 ^a^	92.60 ± 1.673 ^b^	42.77 ± 0.623 ^d^	46.20 ± 2.689 ^c^

Values are mean ± standard deviation, results with different superscripts in the same row mean that there is statistical difference (*p* < 0.05). WPI: whey protein isolate, the differences in percentage in the samples correspond to corn starch.

## Data Availability

The raw data supporting the conclusions of this article will be made available by the authors on request.
